# Health behavior interventions for university students measuring mental health outcomes: A scoping review

**DOI:** 10.3389/fpubh.2022.1063429

**Published:** 2022-12-07

**Authors:** Melinda J. Hutchesson, Megan C. Whatnall, Nazish Yazin, Sasha Fenton, Mitch J. Duncan, Frances J. Kay-Lambkin, Tracy L. Burrows

**Affiliations:** ^1^School of Health Sciences, College of Health, Medicine and Wellbeing, University of Newcastle, Callaghan, NSW, Australia; ^2^School of Medicine and Public Health, College of Health, Medicine and Wellbeing, University of Newcastle, Callaghan, NSW, Australia

**Keywords:** university student (MeSH), health behavior (MeSH), scoping review, mental health, intervention

## Abstract

**Introduction:**

Many university students have poor mental health, and co-occurring health risk behaviors. Targeting health behavior change in this population may improve mental health outcomes. This scoping review describes the extent and range of randomized controlled trials (RCT) evaluating interventions targeting health risk behaviors and measuring a mental health outcome, among university students.

**Methods:**

Six electronic databases were searched for RCTs published until the 18^th^ May 2021. Eligible RCTs included university students, evaluated interventions that promoted health behavior change (i.e., dietary intake, physical activity, sedentary behavior, alcohol and drug use, smoking, and sleep), and measured a mental health-related outcome.

**Results:**

Fifty-nine RCTs met the inclusion criteria that were published from 2000 to 2021, and over half (*n* = 33) were conducted in the United States. Interventions evaluated within the RCTs (*n* = 92) predominantly targeted changes to dietary intake (*n* = 41 interventions), physical activity (*n* = 39), or alcohol intake (*n* = 35). Most interventions targeted one (*n* = 51) or two (*n* = 27) health behaviors only. Included RCTs considered mental ill health outcomes (*n* = 24), psychological wellbeing outcomes (*n* = 20), or both (*n* = 15).

**Discussion:**

This scoping review identified a moderate volume of experimental research investigating the impact of health behavior interventions on university students' mental health. There is scope for further research examining health behavior interventions targeting university students, particularly interventions taking a multi-behavioral approach.

## Introduction

A substantial proportion of university students experience mental health disorders and low psychological well-being (1–5), and research demonstrates a higher prevalence in university students compared with the broader young adult population (18–24 years) (1, 5). Internationally, 31% of university students reported symptoms of one or more of the following conditions–anxiety, mood or substance disorder–in the last 12 months (6). Data from Australian and Italian surveys report that 36–65% of tertiary students experienced high or severe psychological distress (1, 7, 8). Mental ill-health and psychological distress adversely influence student participation, engagement, and performance, and can also negatively affect longer-term outcomes such as employment, income and relationships (2, 6, 9).

Risk factors for mental ill health include health behaviors such as poor diet quality, physical inactivity, sedentary behavior, alcohol and drug use, smoking, and inadequate sleep quantity and quality (1, 3, 8, 10–13). These health behaviors are associated with mental health when examined as separate risk factors and in combination (14, 15). For example, meta-analyses have inferred smoking as a causal factor in the onset of depression, schizophrenia and bipolar disorder (11, 16), and poor sleep as a causal factor in bipolar disorder and a risk factor for suicidal behavior (11, 17). A significant proportion of university students report health risk behaviors (8, 11). The 2021 National College Health Assessment surveys (*n* = 96,489) reported that 64% of college students consumed <3 serves of vegetables per day, 58% were not meeting physical activity recommendations, 17% were current smokers, and 41% were not meeting sleep duration recommendations (18). In Australia, data indicate that 54% of students consumed <3 serves of vegetables per day, 29% were not meeting physical activity guidelines, seven percent were current smokers, and 23% were not meeting sleep duration recommendations (3). These poor lifestyle behaviors expose university students to greater risk of mental health disorders and psychological distress (11).

Given the high prevalence of mental ill health and psychological distress (5), and health risk behaviors among university students (8, 11), targeting behavior-change in this population is important, as improvements in health behaviors (e.g., smoking, sleep, alcohol consumption) may mediate improvements in mental health (11, 19). Universities and other tertiary institutions are well positioned to support students to adopt healthier behaviors and have the potential to reach large numbers of students (19–21). A plethora of interventions aimed at improving diet, physical activity, sedentary behavior, alcohol and drug use, smoking, and sleep in university students have been conducted with varying levels of effectiveness reported (19, 21–23). Further, a recent systematic umbrella review located 17 reviews that focused on the effectiveness of interventions to improve university students' mental health (21), but none of these specifically evaluated the effect of health behavior interventions (21). Therefore, while numerous studies have investigated interventions aimed at improving health behaviors in university students, and numerous studies have investigated interventions aimed at improving mental health in university students, to our knowledge no review has been conducted on the extent and range of experimental research that has targeted health behavior change and measured mental health outcomes.

To address this gap in the literature, the aim of this scoping review is to describe the extent and range of randomized controlled trials (RCT) that evaluate interventions targeting health risk behaviors (i.e., dietary intake, physical activity, sedentary behavior, alcohol and drug use, smoking, and sleep) and measure a mental health outcome, among university students.

## Methods

### Protocol and registration

The methods undertaken in this review align with the PRISMA-ScR (Preferred Reporting Items for Systematic Reviews and Meta-Analyses extension for Scoping Reviews) guidelines (24). The review protocol was prospectively registered with Open Science Framework (25).

### Eligibility criteria

The eligibility criteria were determined using the Population-Intervention-Comparison-Outcome-Study design (PICOS) format. Participants were university students enrolled in a tertiary education institution, namely a “university” or “college.” This also included those students enrolled in vocational education or equivalent. Interventions that were behavioral interventions designed to target one or more health behaviors and implemented within a tertiary education setting were deemed eligible. A behavioral intervention was defined as a coordinated set of activities designed to change specified behavior patterns (26). Interventions of interest were those that were single or multiple behavioral interventions, developed to positively change one or more of the health behaviors i.e., dietary intake or eating behaviors, physical activity, sedentary behavior, alcohol intake, sleep, smoking status, or drug use. Any comparator or control was considered for inclusion. This extended to no intervention or usual care control groups and/or another active intervention group. Studies where the primary or secondary outcome was a mental health outcome, either psychological well-being or mental health disorder-related outcomes were included. Psychological well-being referred to hedonic (e.g., happiness, positive emotions) and eudemonic (e.g., self-acceptance, autonomy) domains. Mental health disorders were described as enclosing depression, anxiety, schizophrenia, bipolar mood disorder, personality disorders and eating disorders (27). Only RCTs were eligible for inclusion.

### Information sources and search

A comprehensive search of six electronic databases was undertaken (MEDLINE, EMBASE, PsycINFO, Web of Science, CINAHL and Cochrane Library) from the date of inception to the 18th May 2021. All searches were limited to records in English, and in human subjects.

The research team developed the search strategy with the University of Newcastle librarian. The search that consisted of focused “text word” searches were utilized appropriately through truncation and indexing to identify articles eligible for inclusion. The complete search strategy is provided in Supplementary Table S1. The reference lists of all included studies were also searched for relevant articles.

### Selection of sources of evidence/study selection

All records from the search, with duplicates removed, were uploaded to covidence, wherein the title and abstracts were screened and full text screening carried out. To minimize bias, two independent reviewers screened the title, abstracts and keywords of all identified records (M. H. and T. B., M.W. N. Y. or S.F.). Records that did not meet one or more of the inclusion criteria were excluded. The full text of records that were deemed relevant, or where reviewers could not determine if eligibility criteria were met from the title, abstract or keywords were retrieved. The full texts were reviewed by two independent reviewers (M. H. and T. B., N. Y, or S.F.). Articles that met all the pre-specified eligibility criteria were included in the scoping review. The articles that did not meet the eligibility criteria had one reason for exclusion recorded. Reasons for exclusion were recorded by the two independent reviewers in a consistent manner. This included reasons for exclusion being recorded in the following order: not a peer reviewed manuscript, incorrect study design, incorrect participants, incorrect setting, incorrect intervention, and incorrect outcome. Where disagreement existed between the two reviewers for inclusion or reasons for exclusion, a third independent reviewer (M. W.) resolved any disagreements.

### Data charting process and data items within the included studies

Data were extracted by one reviewer (N. Y.) and checked by another reviewer (S.F.). The data collection form was developed by the review team for the purposes of the review, and pilot tested prior to implementation using five of the included studies. The extracted data included study characteristics (author, publication date, country), participant characteristics (e.g., sample size, setting, age range, sex), study intervention (e.g., type of health risk behavior(s) targeted by the intervention(s), and comparator and intervention duration), study outcomes (e.g., type of mental health outcomes—psychological well-being or mental health disorders, and whether it was a primary or secondary outcome and follow-up timepoints).

### Synthesis of results

The results are presented in a narrative summary to elucidate the extent and nature of studies for each data item extracted (28). Results are presented by study characteristics (author, year of publication, country, study design), participants criteria (total number of participants, gender/sex, student and mental health-related inclusion criteria) intervention and comparator characteristics (number of study and intervention arms, number and types of health behaviors targeted, intervention duration) and mental health outcome(s) (psychological well-being and/or mental ill health).

## Results

**Figure 1:**
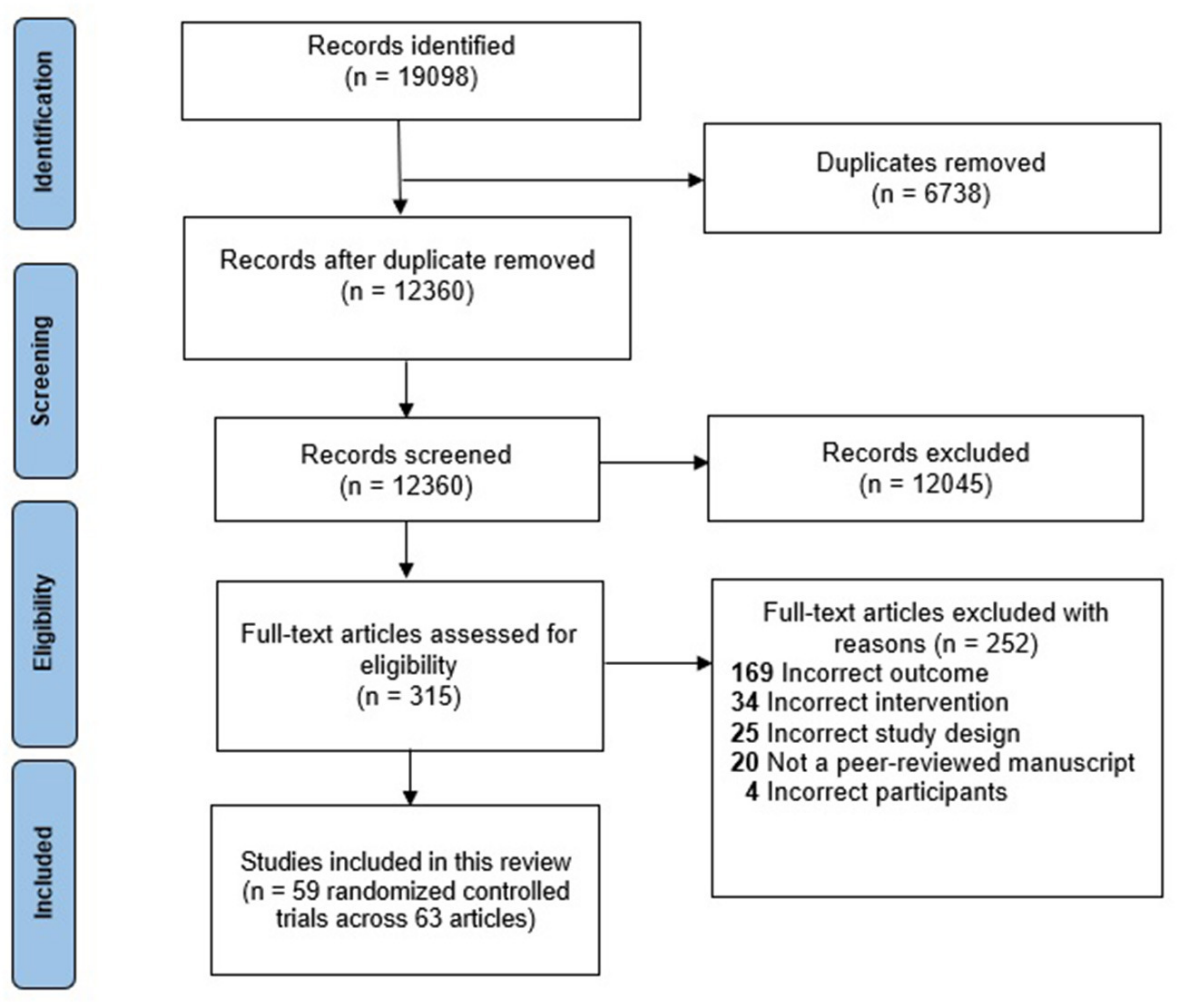
Flow diagram of included studies.

Of 12,360 articles screened based on their title and abstracts, 315 full-text articles were assessed for eligibility. Sixty-three articles were included in the scoping review (29–89), which reported on 59 RCTs (Figure 1) (29–79, 81–84, 86–89). Of the 252 articles excluded from the scoping review, 20 were excluded as they were not peer-reviewed manuscripts, 25 were not RCTs, four did not include tertiary education students, 34 did not evaluate a relevant health behavior intervention, and 169 did not assess a mental health outcome

Table 1 describes the study characteristics and inclusion criteria of the 59 included RCTs and describes the studies by outcomes measured (i.e., psychological well-being only, mental ill health only, and studies with both psychological well-being and mental ill health as outcomes) (29–79, 81–84, 86–89). Supplementary Table S2 provides full details of the individual RCTs (29–79, 81–84, 86–89).

**Table 1:** Summary of study characteristics of 59 RCTs evaluating interventions targeting various health risk behaviors in university students.

		**All studies *n* (%)**	**Psychological well-being only** ***n*** **(%)**	**Mental ill health only** ***n*** **(%)**	**Both** ***n*** **(%)**
Country	United States	33 (55.9)	9 (45.0)	15 (62.5)	9 (60.0)
	United Kingdom	5 (8.5)	1 (5.0)	3 (12.5)	1 (6.7)
	Canada	5 (8.5)	1 (5.0)	2 (8.3)	2 (13.3)
	Australia	4 (6.8)	3 (15.0)	0 (0.0)	1 (6.7)
	Other	12 (20.3)	6 (30.0)	4 (16.7)	2 (13.3)
Publication year	2000-2011	12 (20.3)	4 (20.0)	6 (25.0)	4 (26.7)
	2012-2017	26 (44.1)	7 (35.0)	14 (58.3)	4 (26.7)
	2018-2021	21 (35.6)	9 (45.0)	4 (16.7)	7 (46.7)
Number of participants	Total	22541	6980	8251	7310
	Mean	382	349	344	487
	Median	152	158	134	160
	Range	18-3755	40-1689	18-2621	29-3755
Participant inclusion criteria:	No gender/sex criteria	51 (86.4)	17 (85.0)	21 (87.5)	13 (86.7)
Gender/Sex	Female only	8 (13.6)	3 (15.0)	3 (12.5)	2 (13.3)
Participant inclusion criteria: Age	Young adults only^a^	21 (35.6)	3 (15.0)	12 (50.0)	6 (40.0)
	Adults aged 18 years and above	38 (64.4)	17 (85.0)	12 (50.0)	9 (60.0)
Participant: Student-related inclusion	Yes	26 (44.1)	12 (60.0)	10 (41.7)	4 (26.7)
criteria	No	33 (55.9)	8 (40.0)	14 (58.3)	11 (73.3)
Participant inclusion criteria:	Yes	17 (28.8)	2 (10.0)	7 (29.2)	8 (53.3)
Mental health	No	42 (71.2)	18 (90.0)	17 (70.8)	7 (46.7)
Study design	RCT	48 (81.4)	17 (85.0)	20 (83.3)	11 (73.3)
	Pilot RCT	9 (15.3)	2 (10.0)	4 (16.7)	3 (20.0)
	Cluster RCT	2 (3.4)	1 (5.0)	0 (0.0)	1 (6.7)
Number of study arms	One	1 (1.7)	0 (0.0)	1 (4.2)	0 (0.0)
	Two	42 (71.2)	15 (75.0)	17 (70.8)	11 (73.3)
	Three	10 (16.9)	2 (10.0)	4 (16.7)	4 (26.7)
	Four	6 (10.2)	3 (15.0)	2 (8.3)	0 (0.0)
Number of intervention arms	One	36 (61.0)	13 (65.0)	14 (58.3)	9 (60.0)
	Two	12 (20.3)	3 (15.0)	5 (20.8)	4 (26.7)
	Three	11 (18.6)	4 (20.0)	5 (20.8)	2 (13.3)
	Total	92	31	39	22
Type of control groups	No intervention	23 (39.0)	7 (35.0)	10 (41.7)	6 (0.40)
	Standard/usual care	16 (27.1)	8 (40.0)	5 (20.8)	3 (20.0)
	Wait-list control	9 (15.3)	2 (10.0)	4 (16.7)	3 (20.0)
	No control group^b^	10 (16.9)	3 (15.0)	5 (20.8)	2 (13.3)
	Unclear	1 (1.7)	0 (0.0)	0 (0.0)	1 (6.7)
Behavioral focus of intervention:	Mean	1.6			
Number of health behaviors of interest	One behavior	51 (55.4)	16 (51.6)	26 (66.7)	10 (45.5)
targeted	Two behaviors	27 (29.3)	12 (38.7)	7 (17.9)	7 (31.8)
	Three behaviors	3 (3.3)	2 (6.4)	0 (0.0)	1 (4.5)
	Four behaviors	2 (2.2)	0 (0.0)	1 (2.6)	1 (4.5)
	Five behaviors	2 (2.2)	1 (3.2)	1 (2.6)	0 (0.0)
	Six behaviors	3 (3.3)	0 (0.0)	0 (0.0)	3 (13.6)
	No behaviors of interest	4 (4.3)	0 (0.0)	4 (10.2)	0
Behavioral focus of intervention:	Diet	41 (44.6)	16 (51.6)	9 (23.1)	10 (43.5)
Type of behavior^c^	Physical activity	39 (42.4)	19 (61.2)	9 (23.1)	13 (59.1)
	Alcohol intake	35 (38.0)	8 (25.8)	17 (43.6)	5 (22.7)
	Sleep	19 (20.7)	3 (9.7)	7 (17.9)	6 (27.3)
	Smoking	10 (10.9)	2 (6.4)	5 (12.8)	1 (4.5)
	Drug use	5 (5.4)	0 (0.0)	2 (5.1)	1 (4.5)
	Sedentary behavior	1 (1.1)	0 (0.0)	0 (0.0)	1 (4.5)
	Other health behaviors	8 (8.7)	0 (0.0)	4 (10.2)	0 (0.0)
Intervention duration	Brief-interventions^d^	13 (22.0)	4 (20.0)	7 (29.2)	2 (13.3)
	1 to < 24 weeks	40 (67.8)	14 (70.0)	15 (62.5)	11 (73.3)
	24 to < 48 weeks	2 (3.4)	1 (5.0)	1 (4.2)	0 (0.0)
	≥48 weeks	2 (3.4)	0 (0.0)	0 (0.0)	2 (13.3)
	Unclear	2 (3.4)	1 (5.0)	1 (4.2)	0 (0.0)
Outcomes: Type of outcome measures	Psychological well-being only	20 (33.9)			
	Mental ill health only	24 (40.7)			
	Both psychological well-being and mental ill health	15 (25.4)			
Outcomes: Mental health as primary	Yes	35 (59.3)			
outcome	No	24 (40.7)			

a Participants age ranged from their late teens to their thirties (approximately ages 17–30 years).

b These included other active intervention arms.

c Values for type of behaviors targeted sum to more than the number of intervention arms as some interventions targeted more than one health behaviors.

d Brief interventions included single delivered sessions that varied in duration ranging from 20 min to 1-h.

**Figure 2:**
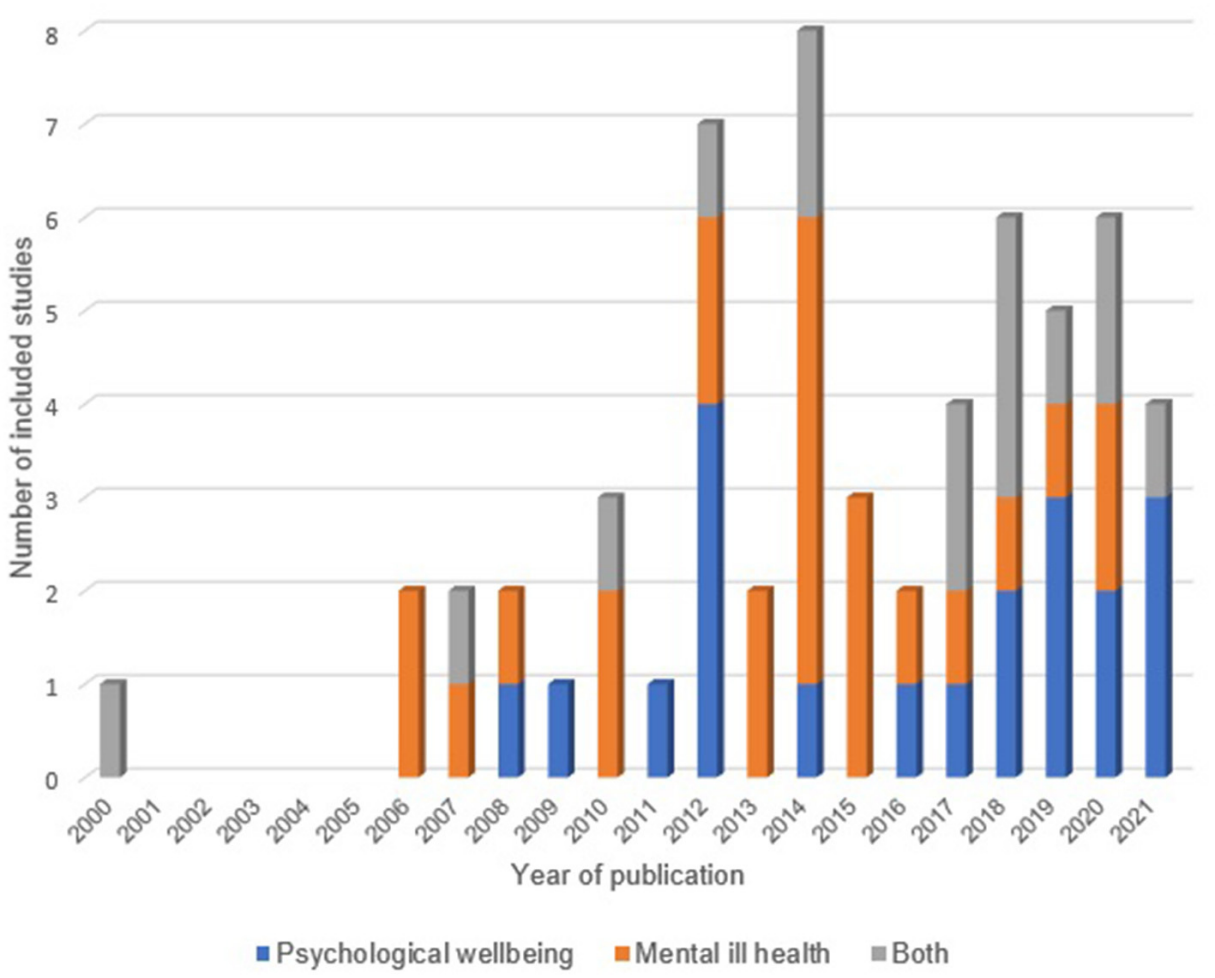
Number of included RCTs per year by type/s of outcome measured (*n* = 59).

The year of publication of the included studies ranged from 2000 to 2021, but only one was published prior to 2006 (Figure 2). Many included studies were published between 2012 and 2017 (*n* = 26, 44.1%) (32, 34, 38, 42, 45, 47–49, 51, 52, 57–59, 64, 65, 67, 69, 71, 73–77, 79, 81, 82). Included studies were conducted in the United States (*n* = 33, 55.9%) (29, 30, 32, 33, 37, 44, 47–51, 55, 57–61, 63–66, 68, 69, 71, 72, 75, 76, 78, 79, 81, 84, 86, 88), the UK (*n* = 5, 8.5%), (34, 42, 45, 52, 67) Canada (*n* = 5, 8.5%) (31, 41, 43, 77, 83), Australia (*n* = 4, 6.8%) (36, 62, 74, 87), Germany (*n* = 2, 3.4%) (46, 56), and nine other countries (*n* = 12, 20.3%).

### Participants

Across the included studies there were a total of 22,541 participants at baseline. The number of participants per study ranged from 18 to 3,755 (Mea*n* = 382, Media*n* = 152). ^(45, 64)^ Over one-third of the studies (*n* = 21, 35.6%) recruited students based on a specified age range (generally young adults aged 17 to 30 years, *n* = 26, 44.1%) with the remaining studies (*n* = 38, 64.1%) recruiting tertiary education students of all ages.

Seventeen studies (28.8%) required particular mental health-related inclusion criteria from the participants (31, 41, 44, 46, 48, 50, 53, 56, 60, 62, 63, 71, 72, 76, 79, 81, 83), e.g., poor psychological well-being or presence or absence of a mental health disorder such as elevated depression or anxiety, severe psychiatric illnesses, diagnosis of an eating disorder, and students undertaking mental health counseling through university services.

Eight (13.6%) studies limited the inclusion criteria by sex (female-only participants) (29, 40, 56, 58, 70, 79, 83, 88). Almost half of the studies (*n* = 26, 44.1%) had further student-related inclusion/exclusion criteria (29, 33–35, 37–40, 42, 47, 49–51, 57, 58, 64, 65, 68, 69, 74, 75, 77, 82, 84, 87, 89), e.g., being a full-time student, undergraduate or graduate student, being a first-year student, and/or students enrolled in a specified university course.

### Interventions and comparator characteristics

As shown in Table 1, most included RCTs included a standard control group (i.e., no intervention, waiting list control or standard care). However, ten (16.9%) studies comparator was another active intervention (e.g., the study compared two different health behavior interventions) (30, 32, 37, 40, 44, 47, 49, 54, 86, 87), and therefore are considered with the description of interventions across the included studies. For each included study, the number of intervention arms ranged between one to four, where 61% (*n* = 36) of the studies evaluated one intervention arm (29, 33, 34, 38, 41–43, 45, 46, 51, 53, 55–58, 60–62, 64, 65, 69–77, 79, 81–84, 87–89). In total, there were 92 intervention arms across the 59 studies. Of the 92 interventions, dietary behaviors were most commonly targeted (*n* = 40, 43.5%) (29, 31, 32, 34, 37, 38, 40–42, 47, 50, 51, 53, 56, 57, 59–62, 70, 71, 75, 78, 79, 82, 84, 86–89) followed by physical activity (*n* = 39, 42.4%) (32, 34, 35, 37, 38, 40, 42, 47, 50, 51, 53, 57, 59, 60, 62, 70, 71, 75, 78, 79, 82, 84, 86, 89), and alcohol intake (*n* = 35, 38%) (34, 36, 42–44, 48, 52, 54, 60, 64–66, 68, 69, 72–74, 83, 84, 86, 87). Sedentary behavior (*n* = 1, 1.1%) was the least targeted behavior (71). Most of the interventions (*n* = 52, 56.5%) targeted improvement in one behavior (e.g., diet, physical activity or alcohol intake only) (29, 30, 33, 35, 36, 39, 40, 43–46, 48–50, 52, 54–56, 58, 61, 63, 65–69, 72–74, 76, 77, 81, 83, 87, 88). Among the studies that targeted two behaviors (*n* = 27, 29.3%) (31, 32, 37, 38, 40, 41, 47, 50, 51, 53, 57, 59, 62, 64, 70, 78, 79, 82), diet and physical activity were the most common combination (23 out of 27 studies). Alcohol intake and smoking were more focused on in the studies that targeted four to six behaviors (33, 42, 60, 84, 86). Most of the interventions (*n* = 40, 67.8%) were conducted for between one and 23 weeks (29–32, 34–36, 38, 41–48, 50–57, 59–63, 65, 67, 75–79, 81, 82, 88, 89). Thirteen (22.0%) were brief interventions; single delivered sessions that varied in duration from 20 min to 1 h (33, 37, 49, 58, 64, 66, 68, 72, 73, 83, 84, 86, 87).

### Outcomes

Of the 59 included studies, 40.7% (*n* = 24) measured mental ill-health outcomes (30, 32, 34, 39, 42, 43, 47–50, 54–56, 58, 64–67, 69, 72, 73, 76, 81, 83), and 33.9% (*n* = 20) measured psychological well-being outcomes (33, 35–37, 40, 51–53, 57, 59, 61, 62, 70, 75, 77, 82, 84, 87–89). One-quarter of the studies (*n* = 15, 25.4%) measured both psychological well-being and mental ill health-related outcomes (29, 31, 38, 41, 44–46, 60, 63, 68, 71, 74, 78, 79, 86). The RCTs measured a variety of mental ill health and psychological well-being outcomes, with the most common outcomes being depression (*n* = 27, 45.8%) (30, 31, 34, 38, 39, 41–43, 46, 48, 49, 55, 58, 60, 63, 65–69, 71–74, 76, 79, 81), anxiety (*n* = 19, 32.2%) (29, 31, 34, 39, 41, 42, 45–47, 49, 58, 60, 63, 67–69, 72, 81, 83), stress (*n* = 17, 28.8%) (31, 37, 41, 44, 46, 51, 53, 57, 59, 61, 62, 68–70, 75, 82, 86), health-related quality of life (*n* = 6, 10.2%) (38, 46, 71, 84, 86, 88), and psychological well-being (*n* = 5, 8.5%) (35, 45, 77, 87, 89).

## Discussion

This scoping review describes the extent and range of RCTs evaluating interventions targeting health behavior change among university students that also measured a mental health-related outcome. The review identified 59 RCTs, with 47 of the RCTs published since 2012. Most interventions focused on a single health behavior, with the three most frequently targeted behaviors including diet, physical activity, and alcohol intake. Of the 41 interventions targeting multiple behaviors, most (*n* = 27) targeted two behaviors and the most frequent combination was diet and physical activity. The mental health focus varied within the included studies, ranging from treatment to prevention of mental ill-health.

This scoping review highlights that most RCTs in this field to date have focused on interventions targeting diet, physical activity, and alcohol intake-related behavior change, with much less focus on sleep, smoking, drug use and sedentary behavior. Given the high prevalence and co-occurrence of health risk behaviors among university students (8, 11), a more equitable distribution across health behaviors would be expected. However, given this scoping reviews specific focus on RCTs that evaluated a mental health outcome, the greater focus on physical activity interventions is somewhat unsurprising given the strong evidence for the role physical activity plays in both the prevention and clinical treatment of mental ill-health (11, 90). The strong focus on dietary behavior change among studies measuring a mental health outcome however is not as well supported by existing evidence, with less certainty of the causal relationship between diet and mental ill-health (11, 91). Notably, the lower number of included RCTs considering sleep is inconsistent with the growing evidence that poor sleep is a risk factor for poor mental health (11, 92–94).

The current scoping review found most RCTs in this field have focused on changing individual health behaviors to influence mental health outcomes. There is strong evidence of co-occurrence of health risk behaviors in the general population (95–97), and specifically among university students (3, 98–103). Further, there is emerging evidence of the association between co-occurrence of health risk behaviors and mental health (3, 98–100). Therefore, more RCTs evaluating multiple health behavior interventions should be anticipated. However, our findings are consistent with the broader multiple health behavior change field, which highlights limited research considering more than two health behaviors, and a predominant focus on diet and physical activity interventions (104). As previously acknowledged, greater multi-disciplinary research and movement beyond individual health behavior silos is required to advance the multiple health behavior research field overall (105), and this is also true for university student-based research.

This scoping review found that many included RCTs evaluating health behavior change interventions, considered mental health as the primary outcome of the study. In addition, included RCTs focused on mental ill-health and/or psychological well-being related outcomes, and almost one-third of studies had an inclusion criterion that considered the mental health status of participants. Collectively, these findings suggest that RCTs conducted to date have considered health behavior change interventions as a strategy for treatment and prevention of mental ill-health, along with mental health promotion. Notably though, a large number of studies (*n* = 151) were excluded from the review as they met all other inclusion criteria except for the requirement to assess a mental health outcome. This emphasizes that despite the evidence of poor mental health among university students, and the association between health behaviors and mental health, many RCTs evaluating health behavior change interventions in this setting have not considered the impact on student's mental health.

Notably, many of the included RCTs limited participants to the young adult population, and often to specific student sub-groups, including first year students/freshman. This focus was supported by the evidence of the transition from school to tertiary education having a negative influence on health behaviors, as well as mental health. Further, many (56%) of the included studies were conducted in the United States, with only a small number (≤ 5) conducted in each of the other countries. Therefore, whilst the results confirm some homogeneity within the available evidence-base, the applicability of the evidence to other student sub-groups (e.g., older students, post-graduate students), and outside the United States, may be limited.

This scoping review has several strengths. It is the first scoping review to comprehensively examine RCTs undertaken to evaluate heath behavior change interventions among university students, with a specific focus on those that measured mental health outcomes. The conduct of the scoping review was consistent with PRISMA-ScR (24), and as such employed a comprehensive search strategy across numerous databases. The study was limited to studies published in English, and therefore may not include all published RCTs. Notably, the review focused on RCTs, as the highest level of evidence from experimental study designs. However, this does mean that other experimental study designs (e.g., non-randomized trials) were excluded from the review. In addition, as is convention for a scoping review the review only considered the extent and range of studies; therefore it did not explore the efficacy of the interventions. Further, the review also did not consider the methodological quality of the included RCTs.

## Conclusion

There is a moderate volume of research exploring the impact of health behavior interventions for university students on mental health outcomes. Of note, the RCTs included in this scoping review can be utilized as a foundation to conduct a systematic review. There is scope for such a systematic review to be limited to specific health behaviors (e.g., diet, physical activity or alcohol intake) or mental health outcomes (e.g., mental ill health or psychological well-being), based on the higher number of included studies. Such a review would help inform the implementation of health behavior interventions in the university setting. Finally, future RCTs examining health behavior interventions targeting university students should: consider less evaluated health behaviors, such as sleep; consider targeting multiple health behaviors within the one intervention approach; and assess mental health as a primary or secondary study outcome.

## Data availability statement

The original contributions presented in the study are included in the article/Supplementary material, further inquiries can be directed to the corresponding author.

## Author contributions

MH: conceptualization, methodology, investigation, writing–original draft, supervision, project administration, and funding acquisition. MW: conceptualization, methodology, investigation, writing–review and editing, supervision, and funding acquisition. NY: methodology, investigation, visualization, writing–review and editing. SF: investigation, visualization, and writing–original draft. MD, FK-L, and TB: conceptualization, methodology, writing-review and editing, and funding acquisition. All authors contributed to the article and approved the submitted version.
