# Supplementary material for: Health behavior interventions for university students measuring mental health outcomes: A scoping review
Source: Front Public Health. 2022 Dec 7;10:1063429. doi: 10.3389/fpubh.2022.1063429 (PMC9771454; doi:10.3389/fpubh.2022.1063429)
Supplement: Supplementary file 1 [file Table_1.DOCX]

Supplementary Table 1 Search Terms by database

Database(s): Ovid MEDLINE(R) and Epub Ahead of Print, In-Process, In-Data-Review & Other Non-Indexed Citations and Daily 1946 to May 14, 2021
Search Strategy:

| **#** | **Searches** | **Results** |
| --- | --- | --- |
| 1 | Universities/ | 43607 |
| 2 | (universit* or college* or polytechnic* or educational institution*).mp. | 525190 |
| 3 | ((higher education or tertiary or undergraduate* or postgraduate* or graduate* or vocational or post secondary or postsecondary) adj5 (campus* or staff* or student* or setting* or population or institution*)).mp. | 57061 |
| 4 | 1 or 2 or 3 | 564505 |
| 5 | Energy Intake/ or Feeding Behavior/ | 120578 |
| 6 | ((energy or micronutrient* or macronutrient* or food or fruit* or vegetable* or fat* or sugar*) adj5 (intake* or consum*)).mp. | 188348 |
| 7 | ((energy or diet*) adj5 (quality or pattern* or behavio* or habit* or intake*)).mp. | 153639 |
| 8 | physical activit*.mp. | 124374 |
| 9 | Exercise/ | 117745 |
| 10 | aerobic.mp. | 92906 |
| 11 | Sedentary Behavior/ | 10713 |
| 12 | ((sedentary or sitting) adj5 (time* or behavio*)).mp. | 17489 |
| 13 | Screen Time/ | 541 |
| 14 | (alcohol* adj5 (intake* or consum* or behavio* or use* or misuse* or abus* or risk* or disorder* or depend*)).mp. | 159983 |
| 15 | Sleep/ | 55974 |
| 16 | alert*.mp. | 43393 |
| 17 | wakeful*.mp. | 25706 |
| 18 | drows*.mp. | 7095 |
| 19 | (sleep* adj5 (quality or tim*)).mp. | 40286 |
| 20 | Cigarette Smoking/ or Smoking/ | 145035 |
| 21 | "Tobacco Use"/ | 2107 |
| 22 | ((smok* or tobacco or cigar*) adj5 (status or use* or behavio*)).mp. | 99127 |
| 23 | Alcohol Drinking/ | 69074 |
| 24 | ((drug* or substance* or cannabis or hallucinogen* or phencyclidine or benzodiazepine or inhal* or opioid* or sedative* or hypnotic or anxiolytic* or stimulant* or amphetamine* or cocaine or marijuana) adj5 (use* or abuse* or misuse* or depend* or disorder*)).mp. | 1208432 |
| 25 | ((health or healthy or lifestyle) adj3 (behavio* or factor*)).mp. | 141257 |
| 26 | 5 or 6 or 7 or 8 or 9 or 10 or 11 or 12 or 13 or 14 or 15 or 16 or 17 or 18 or 19 or 20 or 21 or 22 or 23 or 24 or 25 | 2241646 |
| 27 | Mental Health/ | 43303 |
| 28 | Self Concept/ | 58073 |
| 29 | "Quality of Life"/ | 209356 |
| 30 | Personal Satisfaction/ | 20124 |
| 31 | Happiness/ | 4582 |
| 32 | Resilience, Psychological/ | 6738 |
| 33 | Depression/ or Stress, Psychological/ | 239225 |
| 34 | Aggression/ | 34356 |
| 35 | Anxiety/ | 87029 |
| 36 | Schizophrenia/ | 103033 |
| 37 | Bipolar Disorder/ | 41561 |
| 38 | Personality Disorders/ | 20428 |
| 39 | "Feeding and Eating Disorders"/ | 15948 |
| 40 | Anorexia/ | 5072 |
| 41 | Bulimia/ | 5548 |
| 42 | ((psychological or mental) adj5 (wellbeing or well being or health or illness* or disorder*)).mp. | 420210 |
| 43 | (wellness or self concept or self esteem or self worth or "quality of life" or satisfaction or happiness or affect or resilience or flourishing or stress or aggression or externali* behavio* or antisocial or prosocial or depression or anxiety or schizophrenia or bipolar or personality disorder* or eating disorder* or anorexia or bulimia or binge eat* or binge disorder*).mp. | 2917965 |
| 44 | 27 or 28 or 29 or 30 or 31 or 32 or 33 or 34 or 35 or 36 or 37 or 38 or 39 or 40 or 41 or 42 or 43 | 3142419 |
| 45 | Randomized Controlled Trial/ | 529672 |
| 46 | controlled clinical trial/ | 94148 |
| 47 | Clinical Trial/ | 528706 |
| 48 | random*.tw. | 1220311 |
| 49 | (control adj group*).tw. | 487134 |
| 50 | 45 or 46 or 47 or 48 or 49 | 1997964 |
| 51 | 4 and 26 and 44 and 50 | 3516 |
| 52 | animals/ not humans/ | 4791692 |
| 53 | 51 not 52 | 3470 |
| **54** | **limit 53 to english language** | **3353** |

Database(s): **APA PsycInfo**1806 to May Week 2 2021
Search Strategy:

| **#** | **Searches** | **Results** |
| --- | --- | --- |
| 1 | colleges/ or College Students/ | 93088 |
| 2 | (universit* or college* or polytechnic* or educational institution*).mp. | 377348 |
| 3 | ((higher education or tertiary or undergraduate* or postgraduate* or graduate* or vocational or post secondary or postsecondary) adj5 (campus* or staff* or student* or setting* or population or institution*)).mp. | 77532 |
| 4 | 1 or 2 or 3 | 415124 |
| 5 | Food Intake/ | 14947 |
| 6 | Eating Behavior/ | 14194 |
| 7 | ((energy or micronutrient* or macronutrient* or food or fruit* or vegetable* or fat* or sugar*) adj5 (intake* or consum*)).mp. | 34114 |
| 8 | ((energy or diet*) adj5 (quality or pattern* or behavio* or habit* or intake*)).mp. | 19128 |
| 9 | physical activit*.mp. | 42590 |
| 10 | Exercise/ or Aerobic Exercise/ | 25803 |
| 11 | Sedentary Behavior/ | 1821 |
| 12 | ((sedentary or sitting) adj5 (time* or behavio*)).mp. | 4153 |
| 13 | Screen Time/ | 430 |
| 14 | (alcohol* adj5 (intake* or consum* or behavio* or use* or misuse* or abus* or risk* or disorder* or depend*)).mp. | 105604 |
| 15 | Sleep/ | 24660 |
| 16 | alert*.mp. | 13230 |
| 17 | wakeful*.mp. | 11542 |
| 18 | drows*.mp. | 2290 |
| 19 | (sleep* adj5 (quality or tim*)).mp. | 21250 |
| 20 | Tobacco Smoking/ | 32942 |
| 21 | ((smok* or tobacco or cigar*) adj5 (status or use* or behavio*)).mp. | 34921 |
| 22 | exp Alcohol Drinking Patterns/ or exp Alcohol Drinking Attitudes/ | 28637 |
| 23 | ((drug* or substance* or cannabis or hallucinogen* or phencyclidine or benzodiazepine or inhal* or opioid* or sedative* or hypnotic or anxiolytic* or stimulant* or amphetamine* or cocaine or marijuana) adj5 (use* or abuse* or misuse* or depend* or disorder*)).mp. | 197648 |
| 24 | ((health or healthy or lifestyle) adj3 (behavio* or factor*)).mp. | 80224 |
| 25 | 5 or 6 or 7 or 8 or 9 or 10 or 11 or 12 or 13 or 14 or 15 or 16 or 17 or 18 or 19 or 20 or 21 or 22 or 23 or 24 | 492178 |
| 26 | Mental Health/ | 71175 |
| 27 | Self-Concept/ | 44110 |
| 28 | "Quality of Life"/ | 42625 |
| 29 | exp Life Satisfaction/ or exp Satisfaction/ or exp Well Being/ | 105817 |
| 30 | Happiness/ | 7935 |
| 31 | "Resilience (Psychological)"/ | 15779 |
| 32 | "Depression (Emotion)"/ | 26031 |
| 33 | Physiological Stress/ | 3258 |
| 34 | Aggressiveness/ | 4458 |
| 35 | Anxiety/ | 65561 |
| 36 | Schizophrenia/ | 88747 |
| 37 | Bipolar Disorder/ | 27948 |
| 38 | Personality Disorders/ | 12474 |
| 39 | Eating Disorders/ | 17137 |
| 40 | Anorexia Nervosa/ | 11554 |
| 41 | Bulimia/ | 7787 |
| 42 | ((psychological or mental) adj5 (wellbeing or well being or health or illness* or disorder*)).mp. | 382815 |
| 43 | (wellness or self concept or self esteem or self worth or "quality of life" or satisfaction or happiness or affect or resilience or flourishing or stress or aggression or externali* behavio* or antisocial or prosocial or depression or anxiety or schizophrenia or bipolar or personality disorder* or eating disorder* or anorexia or bulimia or binge eat* or binge disorder*).mp. | 1328704 |
| 44 | 26 or 27 or 28 or 29 or 30 or 31 or 32 or 33 or 34 or 35 or 36 or 37 or 38 or 39 or 40 or 41 or 42 or 43 | 1536008 |
| 45 | Randomized Controlled Trials/ | 730 |
| 46 | clinical trials/ | 11909 |
| 47 | randomized clinical trials/ | 221 |
| 48 | random*.tw. | 213484 |
| 49 | (control adj group*).tw. | 86870 |
| 50 | 45 or 46 or 47 or 48 or 49 | 281199 |
| 51 | 4 and 25 and 44 and 50 | 1671 |
| 52 | animals/ not humans/ | 7305 |
| 53 | 51 not 52 | 1670 |
| **54** | **limit 53 to english language** | **1590** |

Database(s): **Embase**1947 to present
Search Strategy:

| **#** | **Searches** | **Results** |
| --- | --- | --- |
| 1 | university/ | 123664 |
| 2 | (universit* or college* or polytechnic* or educational institution*).tw. | 917359 |
| 3 | ((higher education or tertiary or undergraduate* or postgraduate* or graduate* or vocational or post secondary or postsecondary) adj5 (campus* or staff* or student* or setting* or population or institution*)).tw. | 71098 |
| 4 | 1 or 2 or 3 | 990272 |
| 5 | caloric intake/ | 66502 |
| 6 | feeding behavior/ | 91647 |
| 7 | ((energy or micronutrient* or macronutrient* or food or fruit* or vegetable* or fat* or sugar*) adj5 (intake* or consum*)).tw. | 218399 |
| 8 | ((energy or diet*) adj5 (quality or pattern* or behavio* or habit* or intake*)).tw. | 164867 |
| 9 | physical activity/ | 172401 |
| 10 | exercise/ | 318968 |
| 11 | aerobic exercise/ | 17696 |
| 12 | sedentary lifestyle/ | 15736 |
| 13 | ((sedentary or sitting) adj5 (time* or behavio*)).tw. | 14433 |
| 14 | screen time/ | 1240 |
| 15 | (alcohol* adj5 (intake* or consum* or behavio* or use* or misuse* or abus* or risk* or disorder* or depend*)).tw. | 219513 |
| 16 | sleep/ | 112045 |
| 17 | alert*.tw. | 66288 |
| 18 | wakeful*.tw. | 18839 |
| 19 | drows*.tw. | 13146 |
| 20 | (sleep* adj5 (quality or tim*)).tw. | 61906 |
| 21 | smoking/ or cigarette smoking/ | 386519 |
| 22 | "tobacco use"/ | 12126 |
| 23 | ((smok* or tobacco or cigar*) adj5 (status or use* or behavio*)).tw. | 129211 |
| 24 | drinking behavior/ | 52369 |
| 25 | ((drug* or substance* or cannabis or hallucinogen* or phencyclidine or benzodiazepine or inhal* or opioid* or sedative* or hypnotic or anxiolytic* or stimulant* or amphetamine* or cocaine or marijuana) adj5 (use* or abuse* or misuse* or depend* or disorder*)).tw. | 584199 |
| 26 | ((health or healthy or lifestyle) adj3 (behavio* or factor*)).mp. | 173210 |
| 27 | 5 or 6 or 7 or 8 or 9 or 10 or 11 or 12 or 13 or 14 or 15 or 16 or 17 or 18 or 19 or 20 or 21 or 22 or 23 or 24 or 25 or 26 | 2214938 |
| 28 | mental health/ | 157300 |
| 29 | self concept/ | 97505 |
| 30 | "quality of life"/ | 506673 |
| 31 | Personal Satisfaction.tw. | 732 |
| 32 | happiness/ | 10265 |
| 33 | resilience.mp. | 36764 |
| 34 | depression/ | 395744 |
| 35 | physiological stress/ | 30408 |
| 36 | aggression/ | 62126 |
| 37 | anxiety/ | 234364 |
| 38 | schizophrenia/ | 192799 |
| 39 | bipolar disorder/ | 58729 |
| 40 | personality disorder/ | 31354 |
| 41 | eating disorder/ | 25793 |
| 42 | anorexia/ | 66142 |
| 43 | bulimia/ | 14594 |
| 44 | ((psychological or mental) adj5 (wellbeing or well being or health or illness* or disorder*)).tw. | 330671 |
| 45 | (wellness or self concept or self esteem or self worth or "quality of life" or satisfaction or happiness or affect or resilience or flourishing or stress or aggression or externali* behavio* or antisocial or prosocial or depression or anxiety or schizophrenia or bipolar or personality disorder* or eating disorder* or anorexia or bulimia or binge eat* or binge disorder*).tw. | 3287759 |
| 46 | 28 or 29 or 30 or 31 or 32 or 33 or 34 or 35 or 36 or 37 or 38 or 39 or 40 or 41 or 42 or 43 or 44 or 45 | 3933818 |
| 47 | randomized controlled trial/ | 658318 |
| 48 | controlled clinical trial/ | 463615 |
| 49 | random*.tw. | 1672556 |
| 50 | (control adj group*).tw. | 715912 |
| 51 | 47 or 48 or 49 or 50 | 2455191 |
| 52 | 4 and 27 and 46 and 51 | 5391 |
| 53 | animal/ not human/ | 1519956 |
| 54 | 52 not 53 | 5383 |
| **55** | **limit 54 to english language** | **5128** |

**CINAHL**

| **#** | **Query** | **Results** |
| --- | --- | --- |
| S1 | (MH "Colleges and Universities") OR (MH "Students, College") OR (MH "College Fraternities and Sororities") | 49,609 |
| S2 | TI ( (universit* or college* or polytechnic* or educational institution*) ) OR AB ( (universit* or college* or polytechnic* or educational institution*) ) | 213,842 |
| S3 | TI ( ((higher education or tertiary or undergraduate* or postgraduate* or graduate* or vocational or post secondary or postsecondary) n5 (campus* or staff* or student* or setting* or population or institution*)) ) OR AB ( ((higher education or tertiary or undergraduate* or postgraduate* or graduate* or vocational or post secondary or postsecondary) n5 (campus* or staff* or student* or setting* or population or institution*)) ) | 28,907 |
| S4 | S1 OR S2 OR S3 | 252,680 |
| S5 | (MH "Energy Intake") | 19,044 |
| S6 | (MH "Eating Behavior") | 17,855 |
| S7 | TI ( ((energy or micronutrient* or macronutrient* or food or fruit* or vegetable* or fat* or sugar*) n5 (intake* or consum*)) ) OR AB ( ((energy or micronutrient* or macronutrient* or food or fruit* or vegetable* or fat* or sugar*) n5 (intake* or consum*)) ) | 49,246 |
| S8 | TI ( ((energy or diet*) n5 (quality or pattern* or behavio* or habit* or intake*)) ) OR AB ( ((energy or diet*) n5 (quality or pattern* or behavio* or habit* or intake*)) ) | 43,893 |
| S9 | "physical activit*" | 81,919 |
| S10 | (MH "Exercise") | 54,957 |
| S11 | (MH "Aerobic Exercises") | 7,123 |
| S12 | TI ( ((sedentary or sitting) n5 (time* or behavio*)) ) OR AB ( ((sedentary or sitting) n5 (time* or behavio*)) ) | 6,378 |
| S13 | (MH "Screen Time") | 507 |
| S14 | TI ( (alcohol* n5 (intake* or consum* or behavio* or use* or misuse* or abus* or risk* or disorder* or depend*)) ) OR AB ( (alcohol* n5 (intake* or consum* or behavio* or use* or misuse* or abus* or risk* or disorder* or depend*)) ) | 59,924 |
| S15 | (MH "Sleep") | 21,132 |
| S16 | "alert*" | 16,720 |
| S17 | (MH "Wakefulness") | 2,147 |
| S18 | "drows*" | 1,576 |
| S19 | TI ( (sleep* n5 (quality or tim*)) ) OR AB ( (sleep* n5 (quality or tim*)) ) | 14,260 |
| S20 | (MH "Smoking") OR ((health or healthy or lifestyle) n3 (behavio* or factor*)) | 746,230 |
| S21 | TI ( ((smok* or tobacco or cigar*) n5 (status or use* or behavio*)) ) OR AB ( ((smok* or tobacco or cigar*) n5 (status or use* or behavio*)) ) | 39,667 |
| S22 | (MH "Alcohol Drinking") OR (MH "Alcohol Drinking in College") OR (MH "Drinking Behavior") | 32,649 |
| S23 | TI ( ((drug* or substance* or cannabis or hallucinogen* or phencyclidine or benzodiazepine or inhal* or opioid* or sedative* or hypnotic or anxiolytic* or stimulant* or amphetamine* or cocaine or marijuana) n5 (use* or abuse* or misuse* or depend* or disorder*)) ) OR AB ( ((drug* or substance* or cannabis or hallucinogen* or phencyclidine or benzodiazepine or inhal* or opioid* or sedative* or hypnotic or anxiolytic* or stimulant* or amphetamine* or cocaine or marijuana) n5 (use* or abuse* or misuse* or depend* or disorder*)) ) | 129,509 |
| S24 | S5 OR S6 OR S7 OR S8 OR S9 OR S10 OR S11 OR S12 OR S13 OR S14 OR S15 OR S16 OR S17 OR S18 OR S19 OR S20 OR S21 OR S22 OR S23 | 1,053,484 |
| S25 | (MH "Mental Health") | 42,317 |
| S26 | (MH "Self Concept") | 33,340 |
| S27 | (MH "Quality of Life") | 118,893 |
| S28 | (MH "Personal Satisfaction") | 13,142 |
| S29 | (MH "Happiness") | 4,603 |
| S30 | (MH "Hardiness") | 12,725 |
| S31 | (MH "Depression") | 113,665 |
| S32 | (MH "Stress, Psychological") | 52,871 |
| S33 | (MH "Aggression") | 11,347 |
| S34 | (MH "Anxiety") | 47,049 |
| S35 | (MH "Schizophrenia") | 26,951 |
| S36 | (MH "Bipolar Disorder") | 12,374 |
| S37 | (MH "Personality Disorders") | 4,679 |
| S38 | (MH "Eating Disorders") OR (MH "Bulimia Nervosa") | 11,253 |
| S39 | (MH "Anorexia") | 1,832 |
| S40 | TI ( ((psychological or mental) n5 (wellbeing or well being or health or illness* or disorder*)) ) OR AB ( ((psychological or mental) n5 (wellbeing or well being or health or illness* or disorder*)) ) | 160,236 |
| S41 | TI ( (wellness or self concept or self esteem or self worth or "quality of life" or satisfaction or happiness or affect or resilience or flourishing or stress or aggression or externali* behavio* or antisocial or prosocial or depression or anxiety or schizophrenia or bipolar or personality disorder* or eating disorder* or anorexia or bulimia or binge eat* or binge disorder*) ) OR AB ( (wellness or self concept or self esteem or self worth or "quality of life" or satisfaction or happiness or affect or resilience or flourishing or stress or aggression or externali* behavio* or antisocial or prosocial or depression or anxiety or schizophrenia or bipolar or personality disorder* or eating disorder* or anorexia or bulimia or binge eat* or binge disorder*) ) | 702,559 |
| S42 | S25 OR S26 OR S27 OR S28 OR S29 OR S30 OR S31 OR S32 OR S33 OR S34 OR S35 OR S36 OR S37 OR S38 OR S39 OR S40 OR S41 | 930,779 |
| S43 | (MH "Randomized Controlled Trials") | 115,405 |
| S44 | (MH "Clinical Trials") | 177,138 |
| S45 | TI random* OR AB random* | 373,812 |
| S46 | TI (control n1 group*) OR AB (control n1 group*) | 115,919 |
| S47 | S43 OR S44 OR S45 OR S46 | 572,260 |
| **S48** | **S4 AND S24 AND S42 AND S47 limited to English and humans** | **2,208** |

**COCHRANE LIBRARY**

| **ID** | **Search** | **Hits** | |
| --- | --- | --- | --- |
| #1 | MeSH descriptor: [Universities] this term only | 946 | |
| #2 | (universit* or college* or polytechnic* or "educational institution*"):ti,ab | 64369 | |
| #3 | ("higher education" or tertiary or undergraduate* or postgraduate* or graduate* or vocational or "post secondary" or postsecondary) near/5 (campus* or staff* or student* or setting* or population or institution*):ti,ab | 5623 | |
| #4 | {OR #1-#3} | 68758 | |
| #5 | MeSH descriptor: [Energy Intake] this term only | 4810 | |
| #6 | MeSH descriptor: [Feeding Behavior] this term only | 3301 | |
| #7 | ((energy or micronutrient* or macronutrient* or food or fruit* or vegetable* or fat* or sugar*) near/5 (intake* or consum*)):ti,ab | 22601 | |
| #8 | ((energy or diet*) near/5 (quality or pattern* or behavio* or habit* or intake*)):ti,ab | 21551 | |
| #9 | "physical activit*":ti,ab | 4 | |
| #10 | MeSH descriptor: [Exercise] this term only | 16464 | |
| #11 | aerobic:ti,ab | 14433 | |
| #12 | MeSH descriptor: [Sedentary Behavior] this term only | 1161 | |
| #13 | ((sedentary or sitting) near/5 (time* or behavio*)) | 3727 | |
| #14 | MeSH descriptor: [Screen Time] this term only | 17 | |
| #15 | (alcohol* near/5 (intake* or consum* or behavio* or use* or misuse* or abus* or risk* or disorder* or depend*)) | 21270 | |
| #16 | MeSH descriptor: [Sleep] this term only | 4011 | |
| #17 | alert*:ti,ab | 5490 | |
| #18 | wakeful*:ti,ab | 1511 | |
| #19 | drows*:ti,ab | 2622 | |
| #20 | (sleep* near/5 (quality or tim*)):ti,ab | 13471 | |
| #21 | MeSH descriptor: [Cigarette Smoking] this term only | 138 | |
| #22 | MeSH descriptor: [Tobacco Use] this term only | 48 | |
| #23 | ((smok* or tobacco or cigar*) near/5 (status or use* or behavio*)) | 13984 | |
| #24 | MeSH descriptor: [Alcohol Drinking] this term only | 3761 | |
| #25 | ((drug* or substance* or cannabis or hallucinogen* or phencyclidine or benzodiazepine or inhal* or opioid* or sedative* or hypnotic or anxiolytic* or stimulant* or amphetamine* or cocaine or marijuana) near/5 (use* or abuse* or misuse* or depend* or disorder*)) or ((health or healthy or lifestyle) near/3 (behavio* or factor*)):ti,ab | 222069 | |
| #26 | {OR #5-#25} | 316426 | |
| #27 | MeSH descriptor: [Mental Health] this term only | 1597 | |
| #28 | MeSH descriptor: [Self Concept] this term only | 2403 | |
| #29 | MeSH descriptor: [Quality of Life] this term only | 24975 | |
| #30 | MeSH descriptor: [Personal Satisfaction] this term only | | 907 |
| #31 | MeSH descriptor: [Happiness] this term only | | 197 |
| #32 | MeSH descriptor: [Resilience, Psychological] this term only | | 259 |
| #33 | MeSH descriptor: [Depression] this term only | | 12730 |
| #34 | MeSH descriptor: [Stress, Psychological] this term only | | 5931 |
| #35 | MeSH descriptor: [Aggression] this term only | | 1223 |
| #36 | MeSH descriptor: [Anxiety] this term only | | 7681 |
| #37 | MeSH descriptor: [Schizophrenia] this term only | | 7601 |
| #38 | MeSH descriptor: [Bipolar Disorder] this term only | | 2725 |
| #39 | MeSH descriptor: [Personality Disorders] this term only | | 576 |
| #40 | MeSH descriptor: [Feeding and Eating Disorders] this term only | | 800 |
| #41 | MeSH descriptor: [Anorexia] this term only | | 397 |
| #42 | MeSH descriptor: [Bulimia] this term only | | 536 |
| #43 | ((psychological or mental) near/5 (wellbeing or well being or health or illness* or disorder*)):ti,ab | | 33529 |
| #44 | (wellness or "self concept" or "self esteem" or "self worth" or "quality of life" or satisfaction or happiness or affect or resilience or flourishing or stress or aggression or "externali* behavio*" or antisocial or prosocial or depression or anxiety or schizophrenia or bipolar or "personality disorder*" or "eating disorder*" or anorexia or bulimia or "binge eat*" or "binge disorder*"):ti,ab | | 307287 |
| #45 | {OR #27-#44} | | 327084 |
| **#46** | **{and #4, #26, #45}** | | **4561** |

**Web of Science**

TI=((universit* or college* or polytechnic* or educational institution*) or ((higher education or tertiary or undergraduate* or postgraduate* or graduate* or vocational or post secondary or postsecondary) and (campus* or staff* or student* or setting* or population or institution*) )) OR AB=((universit* or college* or polytechnic* or educational institution*) or ((higher education or tertiary or undergraduate* or postgraduate* or graduate* or vocational or post secondary or postsecondary) and (campus* or staff* or student* or setting* or population or institution*) ))

TI=(“energy intake” or “feeding behavio*” or ((energy or micronutrient* or macronutrient* or food or fruit* or vegetable* or fat* or sugar*) and (intake* or consum*) ) or ((energy or diet*) and (quality or pattern* or behavio* or habit* or intake*) ) or “physical activit*” or Exercise or aerobic or “Sedentary Behavio*” or ((sedentary or sitting) and (time* or behavio*) ) or “Screen Time” or (alcohol* and (intake* or consum* or behavio* or use* or misuse* or abus* or risk* or disorder* or depend*) ) or sleep or alert* or wakeful* or drows* or Smoking or ((smok* or tobacco or cigar*) and (status or use* or behavio*) ) or “Alcohol Drinking” or ((drug* or substance* or cannabis or hallucinogen* or phencyclidine or benzodiazepine or inhal* or opioid* or sedative* or hypnotic or anxiolytic* or stimulant* or amphetamine* or cocaine or marijuana) and (use* or abuse* or misuse* or depend* or disorder*) ) and ((health or healthy or lifestyle) and (behavio* or factor*) )) OR AB=(“energy intake” or “feeding behavio*” or ((energy or micronutrient* or macronutrient* or food or fruit* or vegetable* or fat* or sugar*) and (intake* or consum*) ) or ((energy or diet*) and (quality or pattern* or behavio* or habit* or intake*) ) or “physical activit*” or Exercise or aerobic or “Sedentary Behavio*” or ((sedentary or sitting) and (time* or behavio*) ) or “Screen Time” or (alcohol* and (intake* or consum* or behavio* or use* or misuse* or abus* or risk* or disorder* or depend*) ) or sleep or alert* or wakeful* or drows* or Smoking or ((smok* or tobacco or cigar*) and (status or use* or behavio*) ) or “Alcohol Drinking” or ((drug* or substance* or cannabis or hallucinogen* or phencyclidine or benzodiazepine or inhal* or opioid* or sedative* or hypnotic or anxiolytic* or stimulant* or amphetamine* or cocaine or marijuana) and (use* or abuse* or misuse* or depend* or disorder*) ) and ((health or healthy or lifestyle) and (behavio* or factor*) ))

TI=(“Mental Health” or "Quality of Life" or ((psychological or mental) and (wellbeing or well being or health or illness* or disorder*) ) or (wellness or “self concept” or “self esteem” or “self worth” or "quality of life" or satisfaction or happiness or affect or resilience or flourishing or stress or aggression or “externali* behavio*” or antisocial or prosocial or depression or anxiety or schizophrenia or bipolar or “personality disorder*” or “eating disorder*” or anorexia or bulimia or “binge eat*” or “binge disorder*”) ) OR AB=(“Mental Health” or "Quality of Life" or ((psychological or mental) and (wellbeing or well being or health or illness* or disorder*) ) or (wellness or “self concept” or “self esteem” or “self worth” or "quality of life" or satisfaction or happiness or affect or resilience or flourishing or stress or aggression or “externali* behavio*” or antisocial or prosocial or depression or anxiety or schizophrenia or bipolar or “personality disorder*” or “eating disorder*” or anorexia or bulimia or “binge eat*” or “binge disorder*”) )

TI=(Random* or trial* or group*)

**Refined by:** **LANGUAGES:** ( ENGLISH ) AND **DOCUMENT TYPES:** ( ARTICLE OR REVIEW )

*Indexes=SCI-EXPANDED, SSCI, A&HCI, CPCI-S, CPCI-SSH, ESCI, CCR-EXPANDED, IC Timespan=All years*

**Scopus**

( TITLE ( universit* OR college* OR polytechnic* OR "educational institution*" OR "higher education" OR tertiary OR undergraduate* OR postgraduate* OR graduate* OR vocational OR "post secondary" OR postsecondary ) AND TITLE-ABS-KEY ( "Mental Health" OR "Quality of Life" OR wellbeing OR "well being" OR health OR illness* OR disorder* OR wellness OR "self concept" OR "self esteem" OR "self worth" OR "quality of life" OR satisfaction OR happiness OR affect OR resilience OR flourishing OR stress OR aggression OR "externali* behavio*" OR antisocial OR prosocial OR depression OR anxiety OR schizophrenia OR bipolar OR "personality disorder*" OR "eating disorder*" OR anorexia OR bulimia OR "binge eat*" OR "binge disorder*" ) AND TITLE ( random* OR trial* OR group* ) AND TITLE-ABS-KEY ( "energy intake" OR "feeding behavio*" OR energy OR micronutrient* OR macronutrient* OR food OR fruit* OR vegetable* OR fat* OR sugar* OR diet* OR "physical activit*" OR exercise OR aerobic OR "Sedentary Behavio*" OR sitting OR "Screen Time" OR alcohol* OR sleep OR alert* OR wakeful* OR drows* OR smok* OR drug* OR substance* OR cannabis OR hallucinogen* OR phencyclidine OR benzodiazepine OR inhal* OR opioid* OR sedative* OR hypnotic OR anxiolytic* OR stimulant* OR amphetamine* OR cocaine OR marijuana OR lifestyle OR "like style*" ” ) ) AND ( LIMIT-TO ( DOCTYPE , "ar" ) OR LIMIT-TO ( DOCTYPE , "re" ) ) AND ( LIMIT-TO ( LANGUAGE , "English" ) )
