# Supplementary material for: Health behavior interventions for university students measuring mental health outcomes: A scoping review
Source: Front Public Health. 2022 Dec 7;10:1063429. doi: 10.3389/fpubh.2022.1063429 (PMC9771454; doi:10.3389/fpubh.2022.1063429)
Supplement: Supplementary file 2 [file Table_2.DOCX]

**Supplementary Table 2** Detailed characteristics of the included studies

| **Author** | **Country of origin** | **Sample size (baseline)** | **Study participants (age range/mean; % female)** | **Type of mental health inclusion criteria** | **Type of student related inclusion criteria** | **Intervention duration (weeks)** | **Number of study arms** | **Control group type** | **Number of intervention arms** | **Behavior Change (Y/N)** | | | | | | | **# of health behaviors targeted** | **Other behaviors targeted** | **Data collection timepoints (weeks)** | **Psychological distress related outcome** | **Mental illness related outcome** | **Mental health related primary outcome** |
| --- | --- | --- | --- | --- | --- | --- | --- | --- | --- | --- | --- | --- | --- | --- | --- | --- | --- | --- | --- | --- | --- | --- |
|  |  |  |  |  |  |  |  |  |  | **Diet** | **P.A^[[1]](#footnote-1)^** | **S.B^[[2]](#footnote-2)^** | **S.L ^[[3]](#footnote-3)^** | **A.I^[[4]](#footnote-4)^** | **S.K^[[5]](#footnote-5)^** | **D.U^[[6]](#footnote-6)^** |  |  |  |  |  |  |
| Whatnall (2019)(1) | Australia | 124 | 17-35/22 yrs;73% female | NA | Excluded if studying outside Australia | NA- brief one off | 2 | NA | 2 | Y | N | N | N | N | N | N | 1 | NA | 0, 13 | Y | N | N |
|  |  |  |  |  |  |  |  |  |  | N | N | N | N | Y | N | N | 1 | NA |  |  |  |  |
| Taylor (2014)(2) | USA | 34 | 18-27/ 20yrs;59% female | excluded if have psychiatric condition | NA | 6 | 2 | WLC | 1 | N | N | N | Y | N | N | N | 1 | NA | 0, 6, 13 | N | Y | N |
| Duan (2017)(3) | Hong Kong | 493 | 17-24/19 yrs; 71% female | NA | Excluded collegiate athletes | 8 | 2 | No intervention | 1 | Y | Y | N | N | N | N | N | 2 | NA | 0, 8, 12 | Y | Y | N |
| Ulla Diez (2012)(4) | Spain, Mexico | 73 | 17-24/18 yrs; 74% female | NA | full time student | 7 | 2 | No intervention | 1 | Y | Y | N | N | N | N | N | 2 | NA | 0, 8, 13 | Y | N | Y |
| Reavley (2014)(5) | Australia | 767 | NA/ 25 yrs; 69% female | NA | Students excluded if planned to study for less than 6 months | 104 | 2 | No intervention | 1 | N | N | N | N | Y | N | N | 1 | NA | 0, 30, 82 | Y | Y | Y |
| Yang (2020)(6) | China | 532 | 16-24/ 20 yrs; 46% female | NA | full time student | 7 | 2 | No intervention | 1 | Y | Y | Y | Y | N | N | N | 3 | internet addiction tendency asssessed by Young’s Diagnostic Questionnaire Diagnostic Questionnaire (YDQ) | 0, 30 | Y | N | Y |
| Werch (2008)(7) | USA | 299 | 18-21/ 19 yrs; 60% female | NA | stuents atending campus medical services center | brief intervention | 2 | standard/usual care | 1 | Y | Y | N | Y | Y | Y | N | 5 | NA | 0, 13, 52 | Y | N | Y |
| Abood (2000)(8) | USA | 70 | NA/ 19 yrs; 100% female | NA | female college athletes | 8 | 2 | No intervention | 1 | Y | N | N | N | N | N | N | 1 | NA | 0, 10 | Y | Y | Y |
| Freeman (2017)(9) | UK | 3755 | ≥18/ NA; NA | NA | NA | 10 | 2 | standard/usual care | 1 | N | N | N | Y | N | N | N | 1 | NA | 0, 3, 10, 22 | Y | Y | Y |
| Abroms (2008)(10) | USA | 83 | 18-24/ 20 yrs; 46% female | NA | NA | 13 | 2 | NA | 2 | N | N | N | N | N | Y | N | 1 | NA | 0, 13, 26 | N | Y | N |
|  |  |  |  |  |  |  |  |  |  | N | N | N | N | N | Y | N | 1 |  |  |  |  |  |
| Merrill (2014)(11) | USA | 330 | 18-25/ 19 yrs; 65% female | NA | freshmen, sophomore or junior in status | 4 | 2 | No intervention | 1 | N | N | N | N | Y | N | N | 1 | NA | 0, 4, 26, 52 | N | Y | Y |
| Annesi (2015)(12) | USA | 98 | NA/ 21 yrs; 56% female | NA | NA | 15 | 3 | NA | 3 | Y | Y | N | N | N | N | N | 2 | NA | UC | N | Y | Y |
|  |  |  |  |  |  |  |  |  |  | Y | Y | N | N | N | N | N | 2 |  |  |  |  |  |
|  |  |  |  |  |  |  |  |  |  | Y | Y | N | N | N | N | N | 2 |  |  |  |  |  |
| Schleicher (2012)(13) | USA | 58 | ≥18/ 21 yrs; 51% female | elevated depressive symptomalogy | NA | 8 | 2 | standard/usual care | 1 | N | N | N | N | N | Y | N | 1 | NA | 0, 8, 12, 24 | N | Y | Y |
| Frith (2017)(14) | USA | 39 | NA/ 23 yrs; 68% female | NA | undergraduate and graduate students | 8 | 2 | NA | 2 | Y | Y | N | N | N | N | N | 2 | NA | 0,8 | N | Y | Y |
|  |  |  |  |  |  |  |  |  |  | Y | Y | N | N | N | N | N | 2 | NA |  |  |  |  |
| Kattelmann (2014)(15) | USA | 1639 | 18-24/ 19 yrs; 67% female | NA | full-time first,second or third year college student, Exclude: being in a major for nutrition, exercise science, and/or health promotion, enrolled in nutriton course | 10 | 2 | WLC | 1 | Y | Y | N | N | N | N | N | 2 | NA | 0, 13, 64 | Y | N | Y |
| Sharp (2016)(16) | Canada | 184 | 17 or older/ 18 yrs; 53% female | NA | full-time undergraduate, graduated high school within past year | 12 | 2 | no intervention | 1 | N | Y | N | N | N | N | N | 1 | NA | 0, 12 | Y | N | Y |
| Stice (2013)(17) | USA | 398 | 17-20/ 18 yrs; 100% female | excluded if current diagnosis of anorexia nervosa, bulimia, binge eating disorder | NA | 4 | 2 | UC | 1 | Y | Y | N | N | N | N | N | 2 | NA | 0, 26, 52, 104 | Y | Y | Y |
| LaChausse (2012)(18) | USA | 358 | NA/NA;66% female | NA | NA | 12 | 3 | No intervention | 2 | Y | Y | N | N | N | N | N | 2 | NA | 0, 14 | Y | N | Y |
|  |  |  |  |  |  |  |  |  |  | Y | Y | N | N | N | N | N | 2 |  |  |  |  |  |
| Geisner (2015)(19) | USA | 311 | 18-24/ 20 yrs; 62% female | depressed mood score of 14 or greater on Beck Depression Inventory-II | NA | 5 | 4 | WLC | 3 | N | N | N | N | Y | N | N | 1 | NA | 0, 9 | N | Y | Y |
|  |  |  |  |  |  |  |  |  |  | N | N | N | N | N | N | N | 0 |  |  |  |  |  |
|  |  |  |  |  |  |  |  |  |  | N | N | N | N | Y | N | N | 1 |  |  |  |  |  |
| Lyzwinski (2019)(20) | Australia | 90 | 18-25/ 20 yrs;67% female | excluded if history of serious psychiatric illness | NA | 11 | 2 | standard/usual care | 1 | Y | Y | N | N | N | N | N | 2 | NA | UC | Y | N | Y |
| Hansson (2006)(21) | Sweden | 82 | NA/ 26 yrs; 71% female | NA | NA | 4 | 3 | NA | 3 | N | N | N | N | Y | N | N | 1 | NA | 0, 52, 104 | N | Y | Y |
|  |  |  |  |  |  |  |  |  |  | N | N | N | N | N | N | N | 0 |  |  |  |  |  |
|  |  |  |  |  |  |  |  |  |  | N | N | N | N | Y | N | N | 1 |  |  |  |  |  |
| Patrick (2014) (22) | USA | 404 | 18-35/22 yrs; 70% female | Excluded if psychiatric condition that prohibited compliance with the study protocol | NA | 104 | 2 | standard/usual care | 1 | Y | Y | Y | N | N | N | N | 3 | NA | 26, 52, 78, 104 | Y | Y | N |
| Friedrich (2018)(23) | Germany | 56 | 19-50/ 26 yrs; 68% female | Excluded if current psychotic episodes | NA | 6 | 2 | WLC | 1 | N | N | N | Y | N | N | N | 1 | NA | 0, 6, 13 | Y | Y | Y |
| Murphy (2012)(24) | USA | 82 | 18-21/ 19 yrs; 50% female | NA | full-time first year students | UC | 2 | No intervention | 1 | N | N | N | N | Y | N | N | 1 | NA | 0, 20 or 30 | N | Y | N |
| Sandrick (2017)(25) | USA | 60 | 18-30/19 yrs; 68% female | NA | full-time students | 8 | 2 | standard/usual care | 1 | Y | Y | N | Y | N | N | N | 3 | NA | 0, 8 | Y | N | Y |
| Pengpid (2013)(26) | South Africa | 152 | >18 / 22 yrs; 13% female | NA | NA | brief intervention | 2 | standard/usual care | 1 | N | N | N | N | Y | N | N | 1 | NA | 0, 26, 52 | N | Y | N |
| Greene  (2012)(27) | USA | 1689 | 18-24/ 19 yrs; 63% female | NA | not majoring in nutrition or exercise science | 10 | 2 | WLC | 1 | Y | Y | N | N | N | N | N | 2 | NA | 0, 13, 65 | Y | N | N |
| Mason (2014)(28) | USA | 18 | 18-23/ 19 yrs; 56% female | NA | students enrolled in undergraduate pscyhology course | brief intervention | 1 | No intervention | 1 | N | N | N | N | Y | N | Y | 2 | NA | 0, 4 | N | Y | N |
| Morris (2016) (29) | UK | 138 | 18-34/21 yrs; 67% female |  | NA | 6 | 3 | WLC | 2 | N | N | N | N | N | N | N | 0 | ICBT for anxiety | 0,7 | N | Y | Y |
|  |  |  |  |  |  |  |  |  |  | N | N | N | Y | N | N | N | 1 | NA |  |  |  |  |
| Wilson (2020)(30) | USA | 132 | 18-30/ 21 yrs; 100% female | NA | NA | 1 | 2 | standard/usual care | 1 | Y | N | N | N | N | N | N | 1 | NA | 0, 1, 4 | Y | N | N |
| Kenney (2014)(31) | USA | 226 | NA/ 18 yrs; 100% female | NA | first-year college women | brief intervention | 2 | standard/usual care | 1 | N | N | N | N | Y | N | N | 1 | NA | 0, 4, 26 | N | Y | N |
| Fleming (2010)(32) | Canada | 986 | ≥18/ 21 yrs; 51% female | NA | NA | 4 | 2 | standard/usual care | 1 | N | N | N | N | Y | N | N | 1 | NA | 0, 26, 52 | N | Y | N |
| Hagger (2012)(33) | UK | 709 | 16-24/ 20 yrs; 58% female | NA | NA | 4 | 4 | No intervention | 3 | N | N | N | N | Y | N | N | 1 | NA | 0,4 | Y | N | N |
|  |  |  |  |  |  |  |  |  |  | N | N | N | N | Y | N | N | 1 |  |  |  |  |  |
|  |  |  |  |  |  |  |  |  |  | N | N | N | N | Y | N | N | 1 |  |  |  |  |  |
| Cameron (2015)(34) | UK | 2621 | 16-24/ 19 yrs; 55% female | NA | First year undergraduate students | 4 | 2 | No intervention | 1 | Y | Y | N | N | Y | Y | N | 4 | NA | 0, 4, 26 | N | Y | N |
| Bowen (2009)(35) | USA | 123 | ≥18/ 20 yrs; 27% female | NA | currently enrolled in psychology course | brief intervention | 2 | standard/usual care | 1 | N | N | N | N | N | Y | N | 1 | NA | 0, 1 | Y | N | N |
| Caudwell (2018)(36) | Australia | 215 | >18/ 21 yrs; 73% female | NA | NA | 4 | 4 | No intervention | 3 | N | N | N | N | Y | N | N | 1 | NA | 0,4 | Y | N | N |
|  |  |  |  |  |  |  |  |  |  | N | N | N | N | Y | N | N | 1 |  |  |  |  |  |
|  |  |  |  |  |  |  |  |  |  | N | N | N | N | Y | N | N | 1 |  |  |  |  |  |
| Werch (2007)(37) | USA | 155 | NA/ 19 yrs; 66% female | NA | NA | brief intervention | 3 | NA | 3 | Y | Y | N | Y | Y | Y | Y | 6 | NA | 0,4 | Y | Y | Y |
|  |  |  |  |  |  |  |  |  |  | Y | Y | N | Y | Y | Y | Y | 6 |  |  |  |  |  |
|  |  |  |  |  |  |  |  |  |  | Y | Y | N | Y | Y | Y | Y | 6 |  |  |  |  |  |
| Stice (2018)(38) | USA | 364 | 17-23/ 19 yrs; 72% female | NA | NA | 6 | 3 | No intervention | 2 | Y | Y | N | N | N | N | N | 2 | NA | 0, 4, 26, 52, 104 | Y | Y | Y |
|  |  |  |  |  |  |  |  |  |  | Y | Y | N | N | N | N | N | 2 |  |  |  |  |  |
| Watt (2006)(39) | Canada | 221 | 17-33/ 19 yrs; 100% female | participants selected based on their ASI scores (Anxiety sensitivity index) | NA | brief intervention | 2 | No intervention | 1 | N | N | N | N | Y | N | N | 1 | NA | 0,10 | N | Y | Y |
| Miller (2020)(40) | USA | 212 | NA/ 20 yrs; 59% female | NA | NA | brief intervention | 4 | No intervention | 3 | N | N | N | N | Y | N | N | 1 | NA | 0,4 | N | Y | Y |
|  |  |  |  |  |  |  |  |  |  | N | N | N | N | Y | N | N | 1 |  |  |  |  |  |
|  |  |  |  |  |  |  |  |  |  | N | N | N | N | N | N | N | 0 |  |  |  |  |  |
| Paulus (2020)(41) | USA | 130 | ≥18/22 yrs; 77% female | included: elevated anxiety sensitivity, score of 17 or greater on AS Index-3; excluded: current participation in mental health treatment | NA | brief intervention | 2 | standard/usual care | 1 | N | N | N | N | Y | N | N | 1 | NA | 0, 1, 4, 13 | N | Y | Y |
| Jacobi (2007)(42) | Germany | 100 | 18-29/ NA;100% female | exclusion: current diagnosis of DSM-IV anorexia nervosa, bulimia nervosa, or eating disorders or purging on a regular basis, had been in treatment for an eating disorder, severe psychopathology e.g. alc or drug problems, use of psychotropic medication, or suicidal actions in the past. | NA | 8 | 2 | WLC | 1 | Y | N | N | N | N | N | N | 1 | NA | 0, 8, 13 | N | Y | Y |
| Halperin (2019)(43) | Puerto Rico | 40 | 18-19/ NA; 72% female | exclude: mentally or physically unable to participate | NA | 10 | 2 | standard/usual care | 1 | Y | Y | N | N | N | N | N | 2 | NA | 0, 10, 26 | Y | N | N |
| Caso (2021)(44) | Italy | 171 | NA/ 20 yrs; 74% female | NA | students attending social psychology course | 8 | 4 | No intervention | 3 | N | Y | N | N | N | N | N | 1 | NA | 0, 8 | Y | N | Y |
|  |  |  |  |  |  |  |  |  |  | N | Y | N | N | N | N | N | 1 |  |  |  |  |  |
|  |  |  |  |  |  |  |  |  |  | N | N | N | N | N | N | N | 0 |  |  |  |  |  |
| Loughran (2018)(45) | USA | 300 | 18-24/ NA; 85% female | NA | NA | 5 | 2 | standard/usual care | 1 | Y | N | N | N | N | N | N | 1 | NA | 0, 6 | Y | N | Y |
| El Morr (2020)(46) | Canada | 160 | ≥18/23 yrs; 79% female | excluded if they indicated episodes of psychotic behaviors during the month prior to the trial | NA | 8 | 2 | WLC | 1 | Y | N | N | Y | N | N | N | 2 | health intimacy | UC | Y | Y | Y |
| Park (2021)(47) | Korea | 46 | NA/NA;100% female | NA | NA | 26 | 2 | standard/usual care | 1 | Y | Y | N | N | N | N | N | 2 | NA | 0,26 | Y | N | N |
| Murphy (2019)(48) | USA | 393 | NA/19 yrs; 61% female | NA | full-time first or second-year student | brief intervention | 3 | No intervention | 3 | N | N | N | N | Y | N | N | 1 | NA | 0, 4, 26, 52, 69 | Y | Y | N |
|  |  |  |  |  |  |  |  |  |  | N | N | N | N | Y | N | N | 1 |  |  |  |  |  |
|  |  |  |  |  |  |  |  |  |  | N | N | N | N | Y | N | N | 1 |  |  |  |  |  |
| Hershner (2018)(49) | USA | 549 | NA/22 yrs;58% female | excluded: taking sleep medications, have psychiatric condition | NA | 8 | 2 | No intervention | 1 | N | N | N | Y | N | N | N | 1 | NA | 0, 1, 8 | N | Y | N |
| Gellis (2013)(50) | USA | 62 | ≥18/ NA; 65% female | NA | students from introductory psychology classes | brief intervention | 2 | NA | 2 | N | N | N | Y | N | N | N | 1 | NA | 0, 4 | N | Y | N |
|  |  |  |  |  |  |  |  |  |  | N | N | N | Y | N | N | N | 1 |  |  |  |  |  |
| Epton (2014)(51) | UK | 1445 | NA/ 19 yrs; 58% female | NA | 1st year undergraduate students | 4 | 2 | no intervention | 1 | Y | Y | N | N | Y | Y | Y | 5 | NA | 0, 4, 26 | N | Y | N |
| Ford (2018)(52) | USA | 29 | 18-22/ 20 yrs; 52% female | Inclusion: past complex trauma history, self-reported traumatic stress symptoms; Exclusion: imminent danger of suicide or inpatient psychiatric  or addiction treatment in the past month | NA | 4 | 2 | NA | 2 | N | N | N | N | Y | N | N | 1 | NA | 0,4 | Y | Y | N |
|  |  |  |  |  |  |  |  |  |  | N | N | N | N | Y | N | N | 1 |  |  |  |  |  |
| Dougall (2011)(53) | USA | 146 | 17-47/21 yrs; 70% female | NA | inclusion: incoming first year and transfer students | brief interventions | 3 | NA | 3 | Y | Y | N | N | N | N | N | 2 | NA | 0, 5, 10, | Y | N | Y |
|  |  |  |  |  |  |  |  |  |  | Y | Y | N | N | N | N | N | 2 |  |  |  |  |  |
|  |  |  |  |  |  |  |  |  |  | Y | Y | N | N | N | N | N | 2 |  |  |  |  |  |
| Loucks (2020)(54) | USA | 96 | 18-28/ 20 yrs; 68% female | Current suicidal ideation, history of bipolar or psychotic disorders or self-injurious behaviours | NA | 9 | 2 | standard/usual care | 1 | Y | Y | N | Y | Y | N | N | 4 | NA | 0,13 | Y | Y | Y |
| Mailey (2010)(55) | USA | 51 | 18-52/25 yrs; 68% female | registered for & receiving mental health counseling services | NA | 10 | 2 | No intervention | 2 | N | Y | N | N | N | N | N | 1 | NA | 0,10 | Y | Y | Y |
| Gow (2010)(56) | USA | 170 | ≤22/ 18 yrs; NA | Excluded if have a psychiatric condition, eg. Schizophrenia | first-year students studying Introduction to Psychology courses | 6 | 4 | No intervention | 3 | Y | Y | N | N | N | N | N | 2 | NA | 0, 7, 13 | N | Y | N |
|  |  |  |  |  |  |  |  |  |  | Y | Y | N | N | N | N | N | 2 |  |  |  |  |  |
|  |  |  |  |  |  |  |  |  |  | N | N | N | N | N | N | N | 0 | NA |  |  |  |  |
| Ehrampoush (2019)(57) | Iran | 104 | 18-20; 19 yrs; 71% female | NA | first year medical students | 39 | 3 | No intervention | 2 | N | N | N | Y | N | N | N | 1 | NA | UC | N | Y | Y |
|  |  |  |  |  |  |  |  |  |  | N | N | N | Y | N | N | N | 1 |  |  |  |  |  |
| El Hakeem Ali (2021)(58) | Egypt | 50 | NA/NA; 100% female | NA | nursing students | UC | 2 | NA | 2 | Y | Y | N | N | N | N | N | 2 | NA | UC | Y | N | Y |
|  |  |  |  |  |  |  |  |  |  | N | Y | N | N | N | N | N | 1 |  |  |  |  |  |
| Ahmad (2020)(59) | Canada | 119 | ≥18/ 25 yrs; 75% female | excluded if indications of psychoses eg. Hallucinations | NA | 8 | 3 | WLC | 2 | Y | N | N | Y | N | N | N | 2 | NA | 0, 4, 8 | Y | Y | Y |
|  |  |  |  |  |  |  |  |  |  | Y | N | N | Y | N | N | N | 2 |  |  |  |  |  |

References

1. Whatnall MC, Patterson AJ, Chiu S, Oldmeadow C, Hutchesson MJ. Feasibility and Preliminary Efficacy of the Eating Advice to Students (EATS) Brief Web-Based Nutrition Intervention for Young Adult University Students: A Pilot Randomized Controlled Trial. Nutrients. 2019;11(4):905.

2. Taylor DJ, Zimmerman MR, Gardner CE, Williams JM, Grieser EA, Tatum JI, et al. A Pilot Randomized Controlled Trial of the Effects of Cognitive-Behavioral Therapy for Insomnia on Sleep and Daytime Functioning in College Students. Behavior Therapy. 2014;45(3):376-89.

3. Duan YP, Wienert J, Hu C, Si GY, Lippke S. Web-Based Intervention for Physical Activity and Fruit and Vegetable Intake Among Chinese University Students: A Randomized Controlled Trial. J Med Internet Res. 2017;19(4):e106.

4. Ulla Díez SM, Fortis AP, Franco SF. Efficacy of a Health-Promotion Intervention for College Students: A Randomized Controlled Trial. Nursing Research. 2012;61(2):121-32.

5. Reavley NJ, McCann TV, Cvetkovski S, Jorm AF. A multifaceted intervention to improve mental health literacy in students of a multicampus university: a cluster randomised trial. Social Psychiatry and Psychiatric Epidemiology. 2014;49(10):1655-66.

6. Yang X-H, Yu H-J, Liu M-W, Zhang J, Tang B-W, Yuan S, et al. The impact of a health education intervention on health behaviors and mental health among Chinese college students. Journal of American College Health. 2020;68(6):587-92.

7. Werch CE, Moore MJ, Bian H, DiClemente CC, Ames SC, Weiler RM, et al. Efficacy of a Brief Image-Based Multiple-Behavior Intervention for College Students. Annals of Behavioral Medicine. 2008;36(2):149-57.

8. Abood DA, Black DR. Health Education Prevention for Eating Disorders Among College Female Athletes. American Journal of Health Behavior. 2000;24(3):209-19.

9. Freeman D, Sheaves B, Goodwin GM, Yu L-M, Nickless A, Harrison PJ, et al. The effects of improving sleep on mental health (OASIS): a randomised controlled trial with mediation analysis. The Lancet Psychiatry. 2017;4(10):749-58.

10. Abroms LC, Windsor R, Simons-Morton B. Getting Young Adults to Quit Smoking: A Formative Evaluation of the X-Pack Program. Nicotine Tob Res. 2008;10(1):27-33.

11. Merrill JE, Reid AE, Carey MP, Carey KB. Gender and depression moderate response to brief motivational intervention for alcohol misuse among college students. J Consult Clin Psychol. 2014;82(6):984-92.

12. Annesi JJ, Howton A, Johnson PH, Porter KJ. Pilot Testing a Cognitive-Behavioral Protocol on Psychosocial Predictors of Exercise, Nutrition, Weight, and Body Satisfaction Changes in a College-Level Health-Related Fitness Course. Journal of American College Health. 2015;63(4):268-78.

13. Schleicher HE, Harris KJ, Campbell DG, Harrar SW. Mood Management Intervention for College Smokers With Elevated Depressive Symptoms: A Pilot Study. Journal of American College Health. 2012;60(1):37-45.

14. Frith E, Loprinzi P. Can Facebook Reduce Perceived Anxiety Among College Students? Randomized Controlled Exercise Trial Using the Transtheoretical Model of Behavior Change. JMIR Ment Health. 2017;4(4):e50.

15. Kattelmann KK, Bredbenner CB, White AA, Greene GW, Hoerr SL, Kidd T, et al. The Effects of Young Adults Eating and Active for Health (YEAH): A Theory-Based Web-Delivered Intervention. Journal of Nutrition Education and Behavior. 2014;46(6):S27-S41.

16. Sharp P, Caperchione C. The effects of a pedometer-based intervention on first-year university students: A randomized control trial. J Am Coll Health. 2016;64(8):630-8.

17. Stice E, Rohde P, Shaw H, Marti CN. Efficacy trial of a selective prevention program targeting both eating disorder symptoms and unhealthy weight gain among female college students. Journal of Consulting and Clinical Psychology. 2012;80(1):164-70.

18. LaChausse RG. My Student Body: Effects of an Internet-Based Prevention Program to Decrease Obesity Among College Students. Journal of American College Health. 2012;60(4):324-30.

19. Geisner IM, Varvil-Weld L, Mittmann AJ, Mallett K, Turrisi R. Brief web-based intervention for college students with comorbid risky alcohol use and depressed mood: Does it work and for whom? Addictive Behaviors. 2015;42:36-43.

20. Lyzwinski LN, Caffery L, Bambling M, Edirippulige S. The Mindfulness App Trial for Weight, Weight-Related Behaviors, and Stress in University Students: Randomized Controlled Trial. JMIR Mhealth Uhealth. 2019;7(4):e12210.

21. Hansson H, Rundberg J, Zetterlind U, Johnsson KO, Berglund M. An intervention program for university students who have parents with alcohol problems: A randomised controlled trial. Alcohol and Alcoholism. 2006;41(6):655-63.

22. Patrick K, Marshall SJ, Davila EP, Kolodziejczyk JK, Fowler JH, Calfas KJ, et al. Design and implementation of a randomized controlled social and mobile weight loss trial for young adults (project SMART). Contemporary Clinical Trials. 2014;37(1):10-8.

23. Friedrich A, Claßen M, Schlarb AA. Sleep better, feel better? Effects of a CBT-I and HT-I sleep training on mental health, quality of life and stress coping in university students: a randomized pilot controlled trial. BMC Psychiatry. 2018;18(1):268.

24. Murphy JG, Dennhardt AA, Skidmore JR, Borsari B, Barnett NP, Colby SM, et al. A randomized controlled trial of a behavioral economic supplement to brief motivational interventions for college drinking. J Consult Clin Psychol. 2012;80(5):876-86.

25. Sandrick J, Tracy D, Eliasson A, Roth A, Bartel J, Simko M, et al. Effect of a Counseling Session Bolstered by Text Messaging on Self-Selected Health Behaviors in College Students: A Preliminary Randomized Controlled Trial. JMIR Mhealth Uhealth. 2017;5(5):e67.

26. Pengpid S, Peltzer K, Van der Heever H, Skaal L. Screening and Brief Interventions for Hazardous and Harmful Alcohol Use among University Students in South Africa: Results from a Randomized Controlled Trial. International Journal of Environmental Research and Public Health. 2013;10(5):2043-57.

27. Greene GW, White AA, Hoerr SL, Lohse B, Schembre SM, Riebe D, et al. Impact of an Online Healthful Eating and Physical Activity Program for College Students. American Journal of Health Promotion. 2012;27(2):e47-e58.

28. Mason M, Benotsch EG, Way T, Kim H, Snipes D. Text Messaging to Increase Readiness to Change Alcohol Use in College Students. The Journal of Primary Prevention. 2014;35(1):47-52.

29. Morris J, Firkins A, Millings A, Mohr C, Redford P, Rowe A. Internet-delivered cognitive behavior therapy for anxiety and insomnia in a higher education context. Anxiety, Stress, & Coping. 2016;29(4):415-31.

30. Wilson RE, Marshall RD, Murakami JM, Latner JD. Brief non-dieting intervention increases intuitive eating and reduces dieting intention, body image dissatisfaction, and anti-fat attitudes: A randomized controlled trial. Appetite. 2020;148:104556.

31. Kenney SR, Napper LE, LaBrie JW, Martens MP. Examining the efficacy of a brief group protective behavioral strategies skills training alcohol intervention with college women. Psychol Addict Behav. 2014;28(4):1041-51.

32. Fleming MF, Balousek SL, Grossberg PM, Mundt MP, Brown D, Wiegel JR, et al. Brief physician advice for heavy drinking college students: a randomized controlled trial in college health clinics. J Stud Alcohol Drugs. 2010;71(1):23-31.

33. Hagger MS, Lonsdale A, Chatzisarantis NLD. A theory-based intervention to reduce alcohol drinking in excess of guideline limits among undergraduate students. British Journal of Health Psychology. 2012;17(1):18-43.

34. Cameron D, Epton T, Norman P, Sheeran P, Harris PR, Webb TL, et al. A theory-based online health behaviour intervention for new university students (U@Uni:LifeGuide): results from a repeat randomized controlled trial. Trials. 2015;16(1):555.

35. Bowen S, Marlatt A. Surfing the urge: Brief mindfulness-based intervention for college student smokers. Psychology of Addictive Behaviors. 2009;23(4):666-71.

36. Caudwell KM, Mullan BA, Hagger MS. Testing an Online, Theory-Based Intervention to Reduce Pre-drinking Alcohol Consumption and Alcohol-Related Harm in Undergraduates: a Randomized Controlled Trial. International Journal of Behavioral Medicine. 2018;25(5):592-604.

37. Werch CEC, Bian H, Moore MJ, Ames S, DiClemente CC, Weiler RM. Brief multiple behavior interventions in a college student health care clinic. J Adolesc Health. 2007;41(6):577-85.

38. Stice E, Rohde P, Shaw H, Gau JM. An experimental therapeutics test of whether adding dissonance-induction activities improves the effectiveness of a selective obesity and eating disorder prevention program. International Journal of Obesity. 2018;42(3):462-8.

39. Watt M, Stewart S, Birch C, Bernier D. Brief CBT for high anxiety sensitivity decreases drinking problems, relief alcohol outcome expectancies, and conformity drinking motives: Evidence from a randomized controlled trial. Journal of Mental Health. 2006;15(6):683-95.

40. Miller MB, Hall N, DiBello AM, Park CJ, Freeman L, Meier E, et al. Depressive symptoms as a moderator of college student response to computerized alcohol intervention. Journal of Substance Abuse Treatment. 2020;115.

41. Paulus DJ, Gallagher MW, Neighbors C, Zvolensky MJ. Computer-delivered personalized feedback intervention for hazardous drinkers with elevated anxiety sensitivity: Study protocol for a randomized controlled trial. Journal of Health Psychology. 2020;26(12):2349-58.

42. Jacobi C, Morris L, Beckers C, Bronisch-Holtze J, Winter J, Winzelberg AJ, et al. Maintenance of internet-based prevention: A randomized controlled trial. International Journal of Eating Disorders. 2007;40(2):114-9.

43. Halperin DT, Laux J, LeFranc-García C, Araujo C, Palacios C. Findings From a Randomized Trial of Weight Gain Prevention Among Overweight Puerto Rican Young Adults. Journal of Nutrition Education and Behavior. 2019;51(2):205-16.

44. Caso D, Capasso M, Oliano D. Using messages targeting psychological versus physical health benefits to promote walking behaviour: A randomised controlled trial. Applied Psychology: Health and Well-being. 2021;13(1):152-73.

45. Loughran T, Schumacher J, Harpel T, Vollmer R. Effectiveness of Intuitive Eating Intervention through a Text Messaging Program among College Students. College student journal. 2018.

46. El Morr C, Ritvo P, Ahmad F, Moineddin R. Effectiveness of an 8-Week Web-Based Mindfulness Virtual Community Intervention for University Students on Symptoms of Stress, Anxiety, and Depression: Randomized Controlled Trial. JMIR Ment Health. 2020;7(7):e18595.

47. Park Y-J, Shin H, Jeon S, Cho I, Park HJ. Development and Effects of College-Based Lifestyle Modification Program for Menstrual Health of Young Adult Women with Irregular Menses: A Randomized Controlled Trial. International Journal of Environmental Research and Public Health. 2021;18(1):233.

48. Murphy JG, Dennhardt AA, Martens MP, Borsari B, Witkiewitz K, Meshesha LZ. A randomized clinical trial evaluating the efficacy of a brief alcohol intervention supplemented with a substance-free activity session or relaxation training. Journal of Consulting and Clinical Psychology,. 2019;87(7):657-69.

49. Hershner S, O'Brien LM. The Impact of a Randomized Sleep Education Intervention for College Students. Journal of Clinical Sleep Medicine. 2018;14(03):337-47.

50. Gellis LA, Arigo D, Elliott JC. Cognitive Refocusing Treatment for Insomnia: A Randomized Controlled Trial in University Students. Behavior Therapy. 2013;44(1):100-10.

51. Epton T, Norman P, Dadzie A-S, Harris PR, Webb TL, Sheeran P, et al. A theory-based online health behaviour intervention for new university students (U@Uni): results from a randomised controlled trial. BMC Public Health. 2014;14(1):563.

52. Ford JD, Grasso DJ, Levine J, Tennen H. Emotion Regulation Enhancement of Cognitive Behavior Therapy for College Student Problem Drinkers: A Pilot Randomized Controlled Trial. Journal of Child & Adolescent Substance Abuse. 2018;27(1):47-58.

53. Dougall AL, Swanson JN, Grimm JR, Jenney CT, Frame MC. Tempering the Decline in College Student Physical Activity Using Informational Interventions: Moderating Effects of Stress and Stage of Change. Journal of Applied Biobehavioral Research. 2011;16(1):16-41.

54. Loucks EB, Nardi WR, Gutman R, Saadeh FB, Li Y, Vago DR, et al. Mindfulness-Based College: A Stage 1 Randomized Controlled Trial for University Student Well-Being. Psychosomatic Medicine. 2021;83(6).

55. Mailey EL, Wójcicki TR, Motl RW, Hu L, Strauser DR, Collins KD, et al. Internet-delivered physical activity intervention for college students with mental health disorders: A randomized pilot trial. Psychology, Health & Medicine. 2010;15(6):646-59.

56. Gow RW, Trace SE, Mazzeo SE. Preventing weight gain in first year college students: An online intervention to prevent the “freshman fifteen”. Eating Behaviors. 2010;11(1):33-9.

57. Ehrampoush MH, Tabei SZ, Mahmoodabad SSM, Fallahzadeh H, Nami M, Khayer E, et al. A study of comparing two cognitive-behavioral workshop for college students: Sleep, wakefulness program and perseverance program. J Family Med Prim Care. 2019;8(3):1222-6.

58. El Hakeem Ali LA, Kader NMA, Mahgoub NA. Dietary/exercise guiding program for improvement of nursing students body image, self-esteem and overweight prevention: A randomized controlled study. Pakistan Journal of Medical and Health Sciences. 2021;15(1):388-92.

59. Ahmad F, El Morr C, Ritvo P, Othman N, Moineddin R. An Eight-Week, Web-Based Mindfulness Virtual Community Intervention for Students’ Mental Health: Randomized Controlled Trial. JMIR Ment Health. 2020;7(2):e15520.

1. P.A refers to physical activity [↑](#footnote-ref-1)
2. S.B refers to sedentary behaviour [↑](#footnote-ref-2)
3. S.L refers to sleep [↑](#footnote-ref-3)
4. A.I refers to alcohol intake [↑](#footnote-ref-4)
5. S.K refers to smoking [↑](#footnote-ref-5)
6. D.U refers to drug use [↑](#footnote-ref-6)
